# Analysis of differentially expressed long non-coding RNAs in LPS-induced human HMC3 microglial cells

**DOI:** 10.1186/s12864-022-09083-6

**Published:** 2022-12-27

**Authors:** Mina Baek, Jin Choul Chai, Hae In Choi, Eunyoung Yoo, Bert Binas, Young Seek Lee, Kyoung Hwa Jung, Young Gyu Chai

**Affiliations:** 1 Department of Molecular and Life Science, Hanyang University, Ansan, 15588 Republic of Korea; 2 Institute of Natural Science and Technology, Hanyang University, Ansan, 15588 Republic of Korea; 3 College of Veterinary Medicine, Seoul National University, Seoul, 08826 Republic of Korea; 4 Department of Bionanotechnology, Hanyang University, Seoul, 04673 Republic of Korea; 5 Department of Biopharmaceutical System, Gwangmyeong Convergence Technology Campus of Korea Polytechnic II, Incheon, 21417 Republic of Korea

**Keywords:** Human microglia, Neuroinflammation, BET inhibitor, JQ1, Long non-coding RNA (LncRNA), RNA sequencing (RNA-seq), Differentially expressed long non-coding RNAs (DElncRNAs), Differentially expressed mRNAs (DEmRNAs)

## Abstract

**Background:**

Long non-coding RNAs (lncRNAs) are emerging as key modulators of inflammatory gene expression, but their roles in neuroinflammation are poorly understood. Here, we identified the inflammation-related lncRNAs and correlated mRNAs of the lipopolysaccharide (LPS)-treated human microglial cell line HMC3. We explored their potential roles and interactions using bioinformatics tools such as gene ontology (GO), kyoto encyclopedia of genes and genomes (KEGG), and weighted gene co-expression network analysis (WGCNA).

**Results:**

We identified 5 differentially expressed (DE) lncRNAs, 4 of which (AC083837.1, IRF1-AS1, LINC02605, and MIR3142HG) are novel for microglia. The DElncRNAs with their correlated DEmRNAs (99 total) fell into two network modules that both were enriched with inflammation-related RNAs. However, treatment with the anti-inflammatory agent JQ1, an inhibitor of the bromodomain and extra-terminal (BET) protein BRD4, neutralized the LPS effect in only one module, showing little or even enhancing effect on the other.

**Conclusions:**

These results provide insight into, and a resource for studying, the regulation of microglia-mediated neuroinflammation and its potential therapy by small-molecule BET inhibitors.

**Supplementary Information:**

The online version contains supplementary material available at 10.1186/s12864-022-09083-6.

## Background

Microglia, the macrophages of the central nervous system (CNS), play crucial roles in its homeostasis and immune defense [1, 2]. In response to inflammatory stimuli such as lipopolysaccharide (LPS), these cells become polarized to the M1 phenotype and produce pro-inflammatory cytokines and oxidative metabolites such as IL-1β, TNF, IL-6, and nitric oxide [3].

The bromodomain and extra-terminal (BET) family proteins are epigenetic readers that control the inflammatory response by regulating the expression of transcription factors and cytokines in T cells, monocytes, and macrophages, and therefore are potential therapeutic targets [4–6]. Small-molecule pan-BET inhibitors such as JQ1 and I-BET151 protected mice against LPS-induced sepsis [7] and attenuated the LPS-stimulated expression of pro-inflammatory genes in murine bone marrow-derived macrophages [8]. Similarly, JQ1 attenuated pro-inflammatory chemokine, cytokine, and interferon response genes in the LPS-treated murine glial cell line BV-2 [9], an observation that we confirmed and expanded in the human microglial cell line HMC3 [10]. Thus, it appears that the BET proteins also regulate neuroinflammation.

Our previous study of LPS/JQ1-treated HMC3 cells [10] determined their transcriptomes by RNA sequencing (RNA-seq), but limited the analysis to differentially expressed mRNAs (DEmRNAs), i.e. to protein-coding genes. However, long non-coding RNAs (lncRNAs), such as lincRNA-Cox2 [11], PACER [12], and THRIL [13], are emerging as additional players in gene regulation [14, 15], including regulation of inflammatory genes [16–18]. Accumulating studies have shown that many lncRNAs, including Lethe [19], NEAT1 [20], AS-IL1α [21], and FIRRE [22], play crucial roles in the immune system by regulating excessive or uncontrolled inflammation.

The present study therefore extends our transcriptomic analysis of the LPS/JQ1-treated HMC3 cells into the category of differentially expressed lncRNAs (DElncRNAs). By re-analyzing the published RNA-seq datasets [10], we identified (i) inflammation-related and BET inhibitor-sensitive DElncRNAs, (ii) their correlated DEmRNAs, and (iii) potential functional networks that contain these two classes of transcripts.

## Results

### Approach

**Fig. 1:**
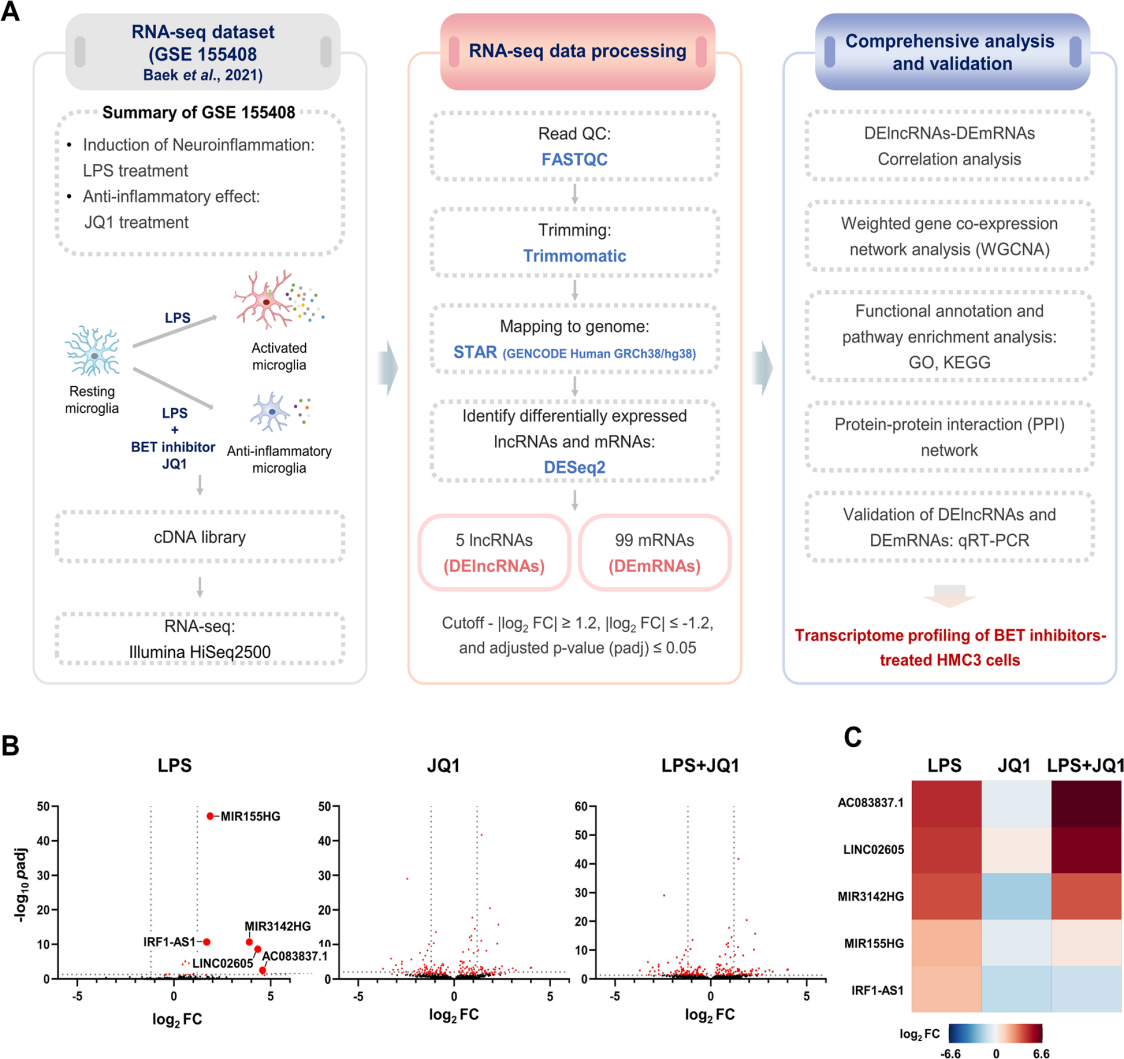
Workflow and identification of DElncRNAs. **A** Schematic summarizing project workflow. **B** Volcano plots visualizing the effects of LPS and/or JQ1 on the lncRNA levels in HMC3 human microglial cells; the treatments were performed in triplicates and the combined results are shown. The gray vertical dot lines indicate log_2_ FC of ≥ 1.2 and ≤ -1.2. The gray horizontal dot lines indicate –log(*p*adj) ≤ 0.01. **C** Heat map showing the expression changes of the 5 LPS-induced lncRNAs that qualified as DElncRNAs by the criteria of this study (see Materials and methods), and the heat maps for the same DElncRNAs upon treatment with JQ1 or LPS + JQ1. The color scale represents the log_2_ FC values. DElncRNAs; differentially expressed lncRNAs, DEmRNAs; differentially expressed mRNAs, log_2_ FC; log_2_ fold change, GO; Gene Ontology, KEGG; Kyoto Encyclopedia of Genes and Genomes

We first identified the LPS-induced DElncRNAs in the dataset GSE155408. Compared to the initial study [10], we added processing methods that are specifically designed to identify DElncRNAs (Fig. 1A); furthermore, we now used a stricter cutoff, which slightly downsized the DEmRNA data set (Supplementary Fig. 1A). We then identified the DEmRNAs that were correlated with the LPS-induced DElncRNAs. In parallel, we assessed whether or how the LPS-induced DE RNAs (including lncRNAs and mRNAs) were affected by JQ1. Next, we performed a network analysis in order to construct interaction modules incorporating the LPS-induced DElncRNAs and their correlated DEmRNAs. This was followed by pathway analysis in order to learn about the potential functional significance of these modules. Finally, we constructed a corresponding protein–protein interaction network.

### Identification of DElncRNAs and DEmRNAs in LPS-treated HMC3 cells

In the control vs. LPS-treated cells, we identified a total of 5 DElncRNAs (all upregulated; Fig. 1B and C) and 99 DEmRNAs (98 upregulated and 1 downregulated; Supplementary Fig. 1A). Conversely, the DElncRNAs and a majority of the DEmRNAs tended to be downregulated or unaffected by JQ1. When LPS and JQ1 were combined, JQ1 further increased the expression of the two DElncRNAs that were most increased by LPS (AC083837.1 and LINC02605), but partially (MIR3142HG) or fully (MIR155HG and IRF1-AS1) neutralized the LPS effect on the other DElncRNAs (Fig. 1C). Likewise, JQ1 counteracted LPS or did not alter its effect for the majority of DEmRNAs, but enhanced the LPS effect in some cases. We note that the above DElncRNAs had not yet been identified in the HMC3 cells; the DEmRNAs are often inflammation- and immunity-related (e.g., CCL20, CSF3, CXCL10, TNF, and CXCL8) (Supplementary Fig. 1B and C). The up- and downregulated DElncRNAs and DEmRNAs are listed in Supplementary Table 1.

### Correlations between DElncRNAs and DEmRNAs

**Fig. 2:**
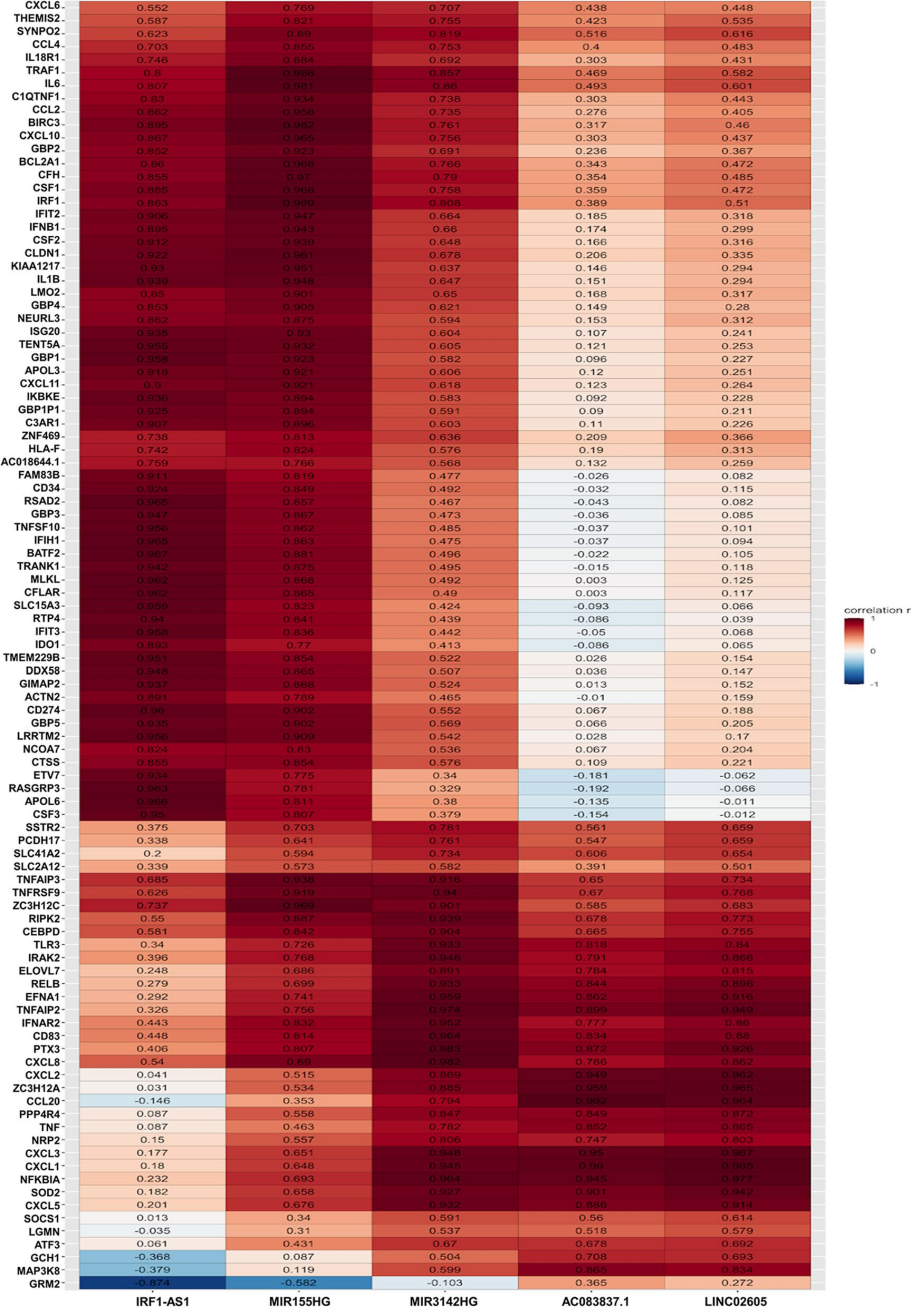
Correlation heat map of the DElncRNAs and DEmRNAs. Each column corresponds to one of the LPS-induced DElnRNAs, and the cells of the rows show for each LPS-induced DEmRNA the Pearson correlation (*r* value) with that given DElncRNA both in color code (according to the color scale shown on the right) and as the numerical value. A red cells indicate the positive correlation, while blue cells indicate the negative correlation, with higher correlation indicated by dark color intensity as shown by the color scale

The heat map in Fig. 2 visualizes the Pearson correlation coefficient (*r*) between the DElncRNAs and DEmRNAs; numerical values are listed in Supplementary Table 2. When including all 5 LPS-induced DElncRNAs and all 99 LPS-induced DEmRNAs into the analysis, a total of 211 DElncRNA-DEmRNA pairs (defined as |*r|*≥ 0.75 and *p*adj ≤ 0.01) were identified. Specifically, MIR155HG, IRF1-AS1, AC083837.1, LINC02605, and MIR3142HG were paired with 73, 57, 36, 21, and 24 DEmRNAs, respectively.

### Identification of DElncRNA-DEmRNA network modules

**Fig. 3:**
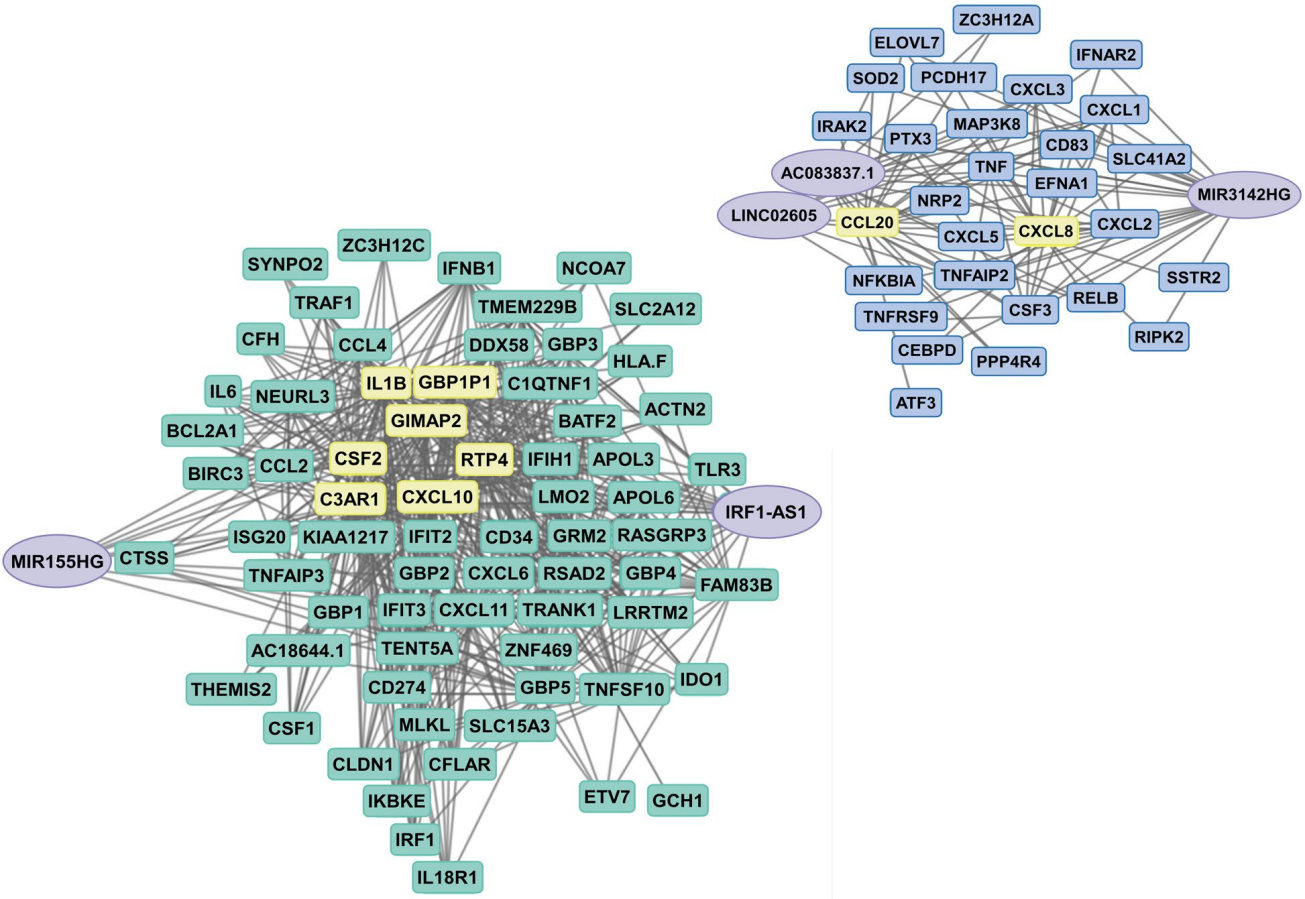
Co-expression networks of DElncRNAs with DEmRNAs. A network was constructed using DElncRNA-DEmRNA pairs with Pearson correlation coefficient (*r*) value ≥ 0.75. We identified 632 connections between the 5 DElncRNAs and 99 DEmRNAs, which could be organized into a large turquoise (left) and a small cyan (right) module. Purple circles denote DElncRNAs, turquoise and cyan rectangles denote DEmRNAs. The yellow rectangles denote DEmRNAs that have core interactions with DElncRNAs. Solid lines indicate correlations

In order to find potential interactions, we constructed co-expression networks based on the above correlations between the DElncRNAs and DEmRNAs. The resulting nodes and relations fell into two modules, which we dubbed “large turquoise” and “small cyan” (Fig. 3). The large turquoise module was defined by IRF1-AS1 and MIR155HG (the two DElncRNAs that were least upregulated by LPS); the small cyan module was defined by AC083837.1, LINC02605, and MIR3142HG (the DElncRNAs that were more strongly induced by LPS). Within these modules, MIR155HG has the maximum number of co-expressed genes.

We determined by chi-square test whether there was a significant difference in how the two modules respond to the JQ1 treatment (Supplementary Table 3). The mRNAs of the large turquoise module were significantly related to JQ1, while the mRNAs of the small cyan module were not related to JQ1. Specifically, out of 65 mRNAs in the large turquoise module, the levels of 64 mRNAs were decreased by JQ1. In contrast, out of 29 mRNAs of the small cyan module, 11 were marginally affected and 18 were increased. In more detail, the mRNA levels of the small cyan module that were highly correlated with MIR3142HG were reduced or marginally affected by JQ1, while the mRNAs that were highly correlated with AC083837.1 and LINC02605 showed increased expression or marginal changes.

**Fig. 4:**
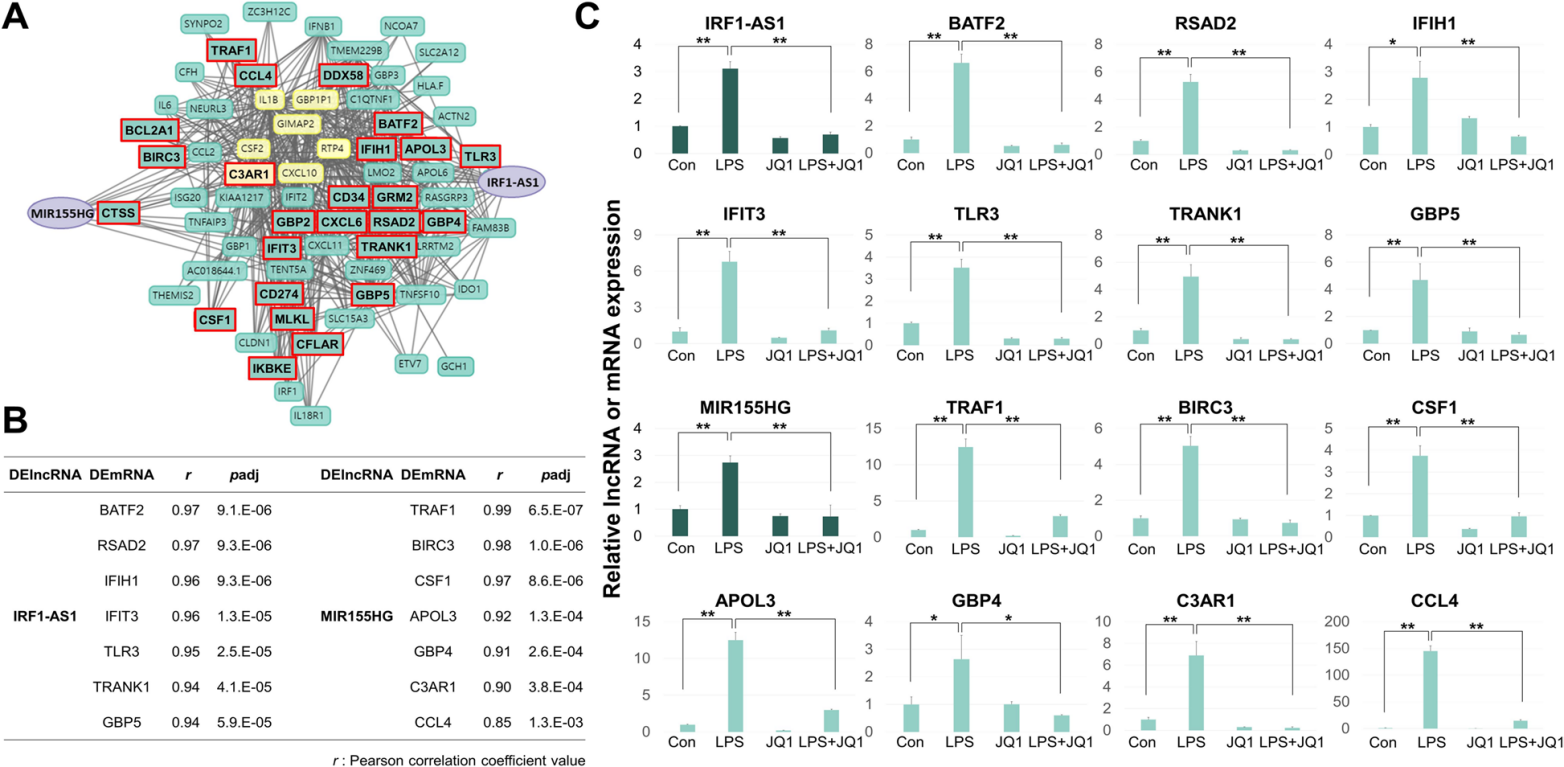
qRT-PCR validation of the large turquoise module. **A** Co-expression network image highlighting the mRNAs of the large turquoise module. Of the genes in the large turquoise module, only selected mRNAs are shown in bold and in red. **B** A list of selected (highly correlated, inflammation-related) DElncRNA-DEmRNA pairs in the large turquoise module. **C** The HMC3 cells were treated with DMSO, LPS, and/or with JQ1, and selected DElncRNAs and DEmRNAs of the large turquoise module assessed by qRT-PCR. The RNA levels are normalized to GAPDH transcript levels. Dark green indicates lncRNA and light green indicates mRNA. The data represent three independent experiments. The values are the mean ± SEM of triplicate experiments (*p < 0.05 and **p < 0.001)

**Fig. 5:**
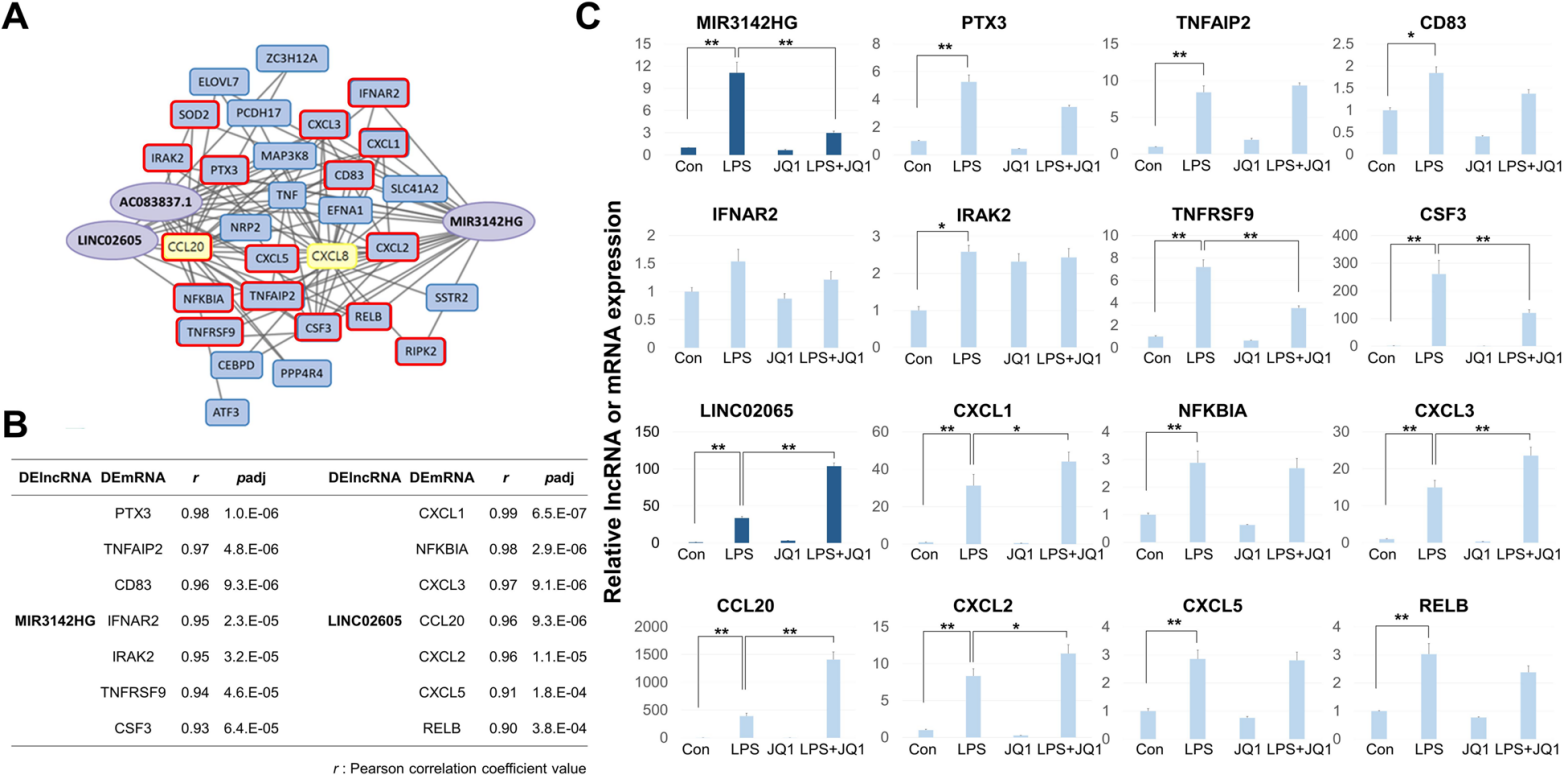
qRT-PCR validation of the small cyan module. **A** Co-expression network image highlighting the mRNAs of the small cyan module. Of the genes in small cyan module, only selected mRNAs are shown in bold and in red. **B** A list of selected DElncRNA-(highly correlated, inflammation-related) DEmRNA pairs in the small cyan module. **C** The HMC3 cells were treated with DMSO, LPS, and/or with JQ1, and selected DElncRNAs and DEmRNAs of the small cyan module assessed by qRT-PCR. The RNA levels are normalized to GAPDH transcript levels. Dark blue indicates lncRNA and light blue indicates mRNA. The data represent three independent experiments. The values are the mean ± SEM of triplicate experiments (*p < 0.05 and **p < 0.001)

To validate the two modules, which were identified on the basis of RNA-seq data, we repeated the LPS/JQ1 treatments of the HMC3 cells and measured the expression levels of the DElncRNAs and selected (i.e., highly correlated, inflammation-related) DEmRNAs. In the large turquoise module, JQ1 mostly neutralized the LPS effect (22 of 23 tested mRNAs, both tested lncRNAs) (Fig. 4 and Supplementary Fig. 2), whereas in the small cyan module, most of the LPS-stimulated RNAs were marginally affected or even further increased by the additional presence of JQ1 (15 of 17 mRNAs, 1 of 2 tested lncRNAs) (Fig. 5 and Supplementary Fig. 2). In this study, primers for qRT-PCR of lncRNAs were designed based on the sequences of IRF1-AS1-203 (ENST00000378953.8), MIR155HG-201 (ENST00000456917.2), and MIR3142HG-201 (ENST00000517927.1) transcripts.

To summarize, while the mRNAs of the large turquoise module mostly were decreased by JQ1, the mRNAs of the small cyan module showed a mixed response pattern.

### Functional annotations

**Fig. 6:**
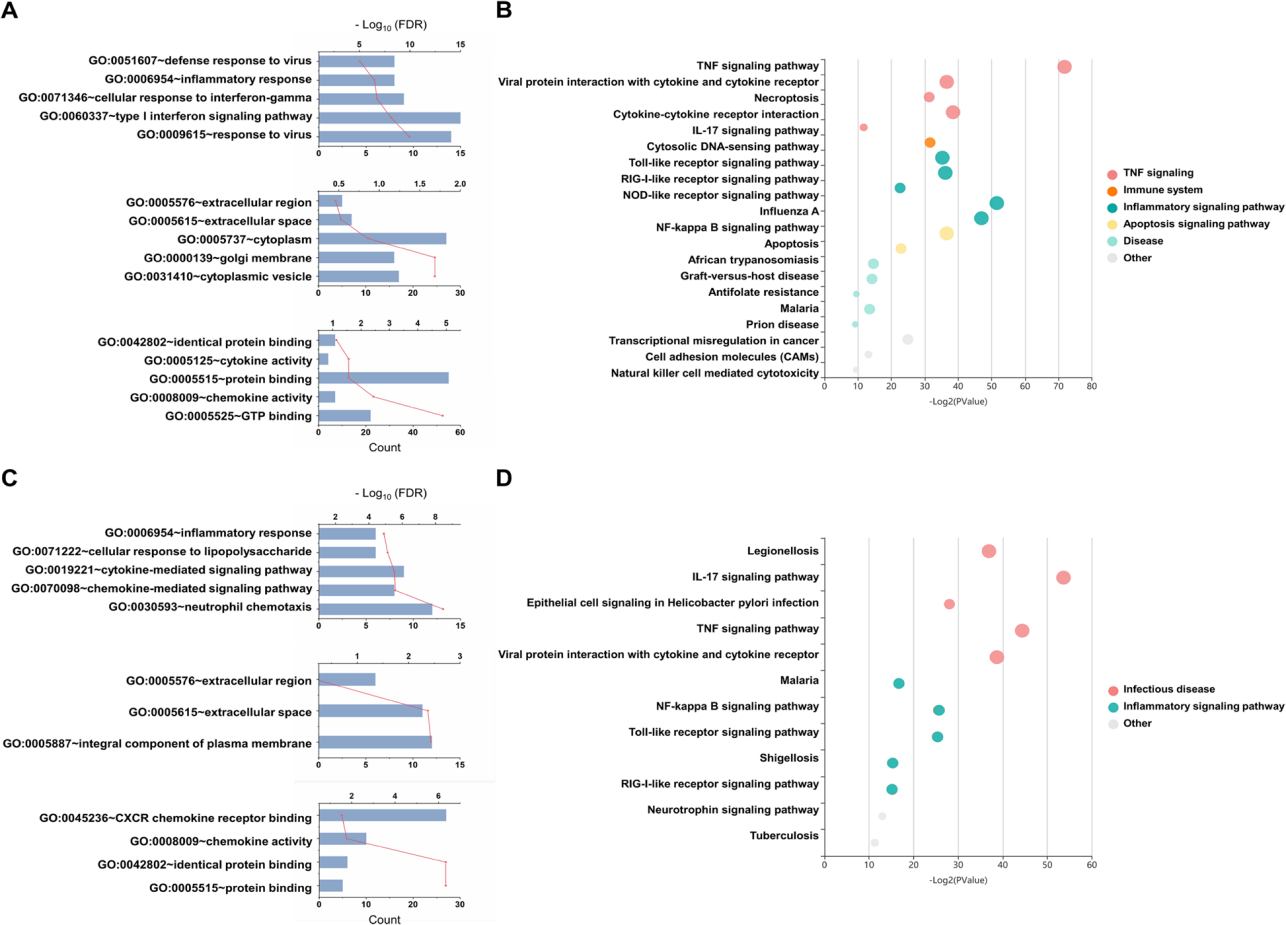
GO and KEGG pathway analyses of the DElncRNAs and DEmRNAs. Shown are the GO term (**A**, **C**) and KEGG pathway enrichment (**B**, **D**) analyses of the large turquoise (**A**, **B**) and small cyan (**C, D**) modules. In the GO term analyses, the numbers of genes and false discovery rate (FDR) values are displayed for the top 5 GO terms in biological process (BP; upper panel), cellular component (CC; middle panel), and molecular function (MF; bottom panel). The blue column is the count value indicating the number of genes enriched in the GO term, and the red line is the -log_10_ (FDR) value. In the KEGG pathway enrichment analyses, each row represents an enriched function, and the size of the bubble represents the *p-*value (KOBAS, http://kobas.cbi.pku.edu.cn). The KOBAS algorithm divides the clusters according to the values computed for the enriched pathway, and the color of each bubble represents a different cluster

Having validated the network modules, we performed a functional classification and pathway enrichment analysis of their respective mRNAs. In both the large turquoise (Fig. 6A) and small cyan (Fig. 6C) modules, Gene Ontology (GO) analysis highlighted terms related to inflammatory response such as type I interferon signaling pathway, defense response to virus, and chemokine-mediated signaling pathway. Similarly, Kyoto Encyclopedia of Genes and Genomes (KEGG) pathway enrichment analysis (Fig. 6B and D) found a total of 32 KEGG pathways (FDR ≤ 0.05, DEmRNA counts ≥ 4) predominantly related to inflammation, such as TNF, IL-17, NOD-like receptor, TLR, and NF-kappa B signaling. The main functions enriched in both modules are related to immune and inflammation. However, we found that apoptosis and cell adhesion-related functions were more enriched in the large turquoise module. The GO and KEGG analysis results of the total mRNAs are shown in Supplementary Fig. 1B and C.

### Protein–protein interaction (PPI) network analysis

**Fig. 7:**
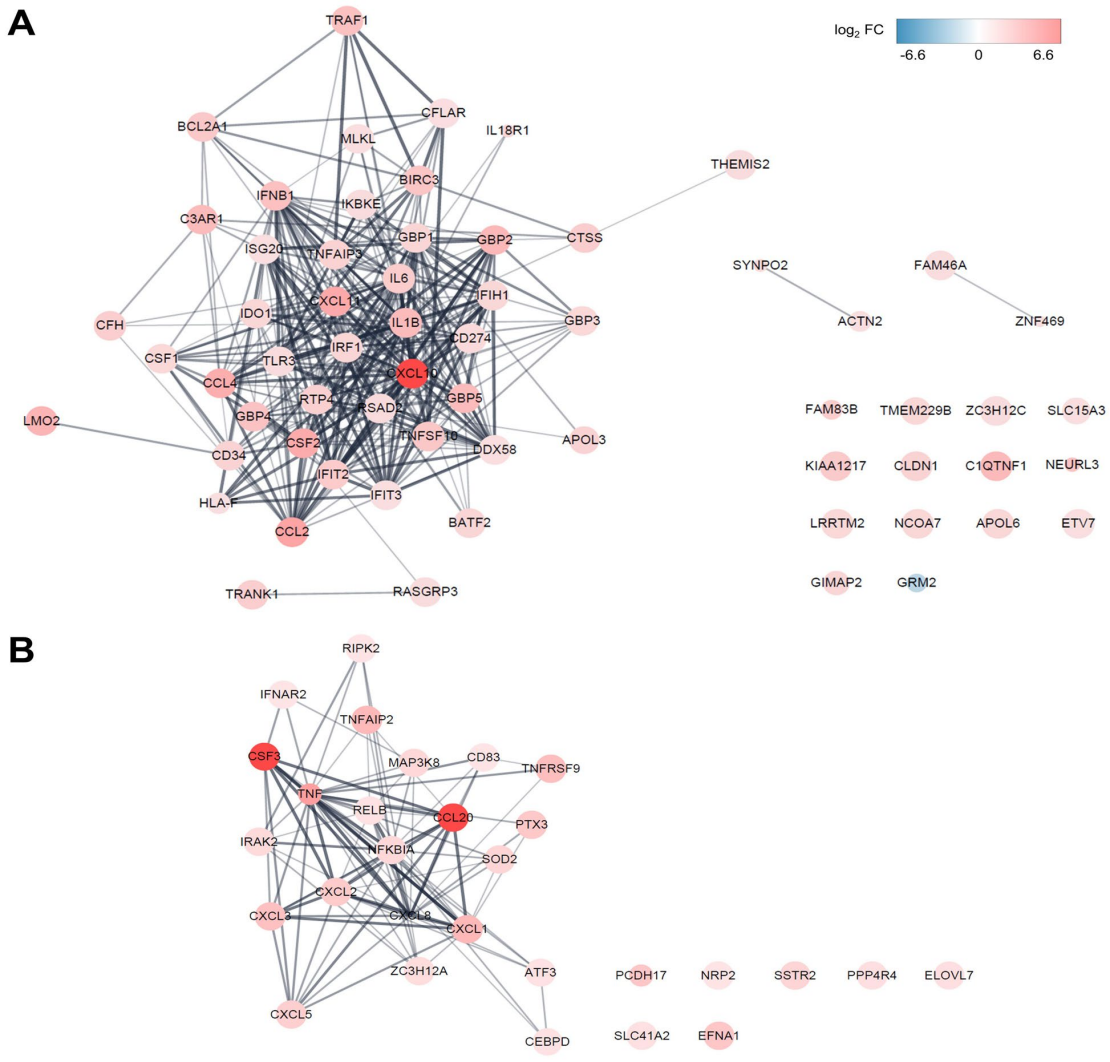
Protein–protein interaction (PPI) network analysis of DEmRNAs. Shown are the PPI analyses of the large turquoise (**A**) and small cyan (**B**) modules. The potential interactions of the proteins encoded by DElncRNA-correlated DEmRNA were determined by STRING online software, using a combined score > 0.7 as cut-off criterion. Lines indicate associations/interactions between genes. The larger the number of connections of a given gene (“node”), the higher the connectivity. A high connectivity indicates the importance of a gene in the PPI network. The color represents the log_2_ FC values and the size of the bubble represents the *p-*value

The PPI network mathematically calculates the physical interactions between proteins in cells, which allowing a molecular assessment at the molecular and system level. We constructed a PPI network of DEmRNAs, which highly correlated DElncRNAs using the STRING database (Fig. 7). The PPI network contained 63 nodes and 332 edges in the co-expressed mRNAs of the large turquoise module (related to the MIR155HG and IRF1-AS1 lncRNAs) and 29 nodes and 90 edges in the co-expressed mRNA of the small cyan module (related to the AC083837.1, LINC02605, and MIR3142HG lncRNAs). The top 5 mRNAs that had the highest node degrees in the large turquoise module were IL1B (degree = 33), IRF1 (degree = 31), IL6 (degree = 29), CXCL10 (degree = 28), and TNFSF10 (degree = 27). The top 5 mRNAs that had the highest node degrees in the small cyan module were TNF (degree = 21), CXCL8 (degree = 18), NFKBIA (degree = 18), CXCL1 (degree = 12), and CXCL2 (degree = 12).

## Discussion

In this study, we identified 5 DElncRNAs in LPS-treated HMC3 human microglial cells. 4 of these transcripts (AC083837.1, IRF1-AS1, LINC02605, and MIR3142HG) have not previously been invoked in the microglial inflammatory response. We further found that the BET inhibitor JQ1 can exert both positive and negative effects on the LPS-induced DElncRNAs. Importantly, we were able to correlate the novel DElncRNAs with inflammation-related DEmRNAs, in line with the idea that the DElncRNAs modulate the microglial immune response. More specifically, we found that the LPS-induced DElncRNAs and their correlated mRNAs fall into two modules that we dubbed “large turquoise” (defined by IRF1-AS1 and MIR155HG) and “small cyan” (defined by AC083837.1, LINC02605, and MIR3142HG). Interestingly, JQ1 affected their patterns in opposite ways: Inflammation processes were decreased by JQ1 in the large turquoise module, but marginally affected or even significantly increased in the small cyan module. We performed qRT-PCR to verify the expression of lncRNAs and mRNAs of the divided modules based on the Pearson correlation coefficient (*r*) of DElncRNAs-DEmRNAs. We found some genes with conflicting DElncRNAs-DEmRNAs correlations as a result of qRT-PCR, which appeared as a probability of false-positive in Pearson's correlation analysis. Pearson correlation analysis is the commonly used analysis method to measure the strength of association in a linear relationship between two datasets based on the mapped read count value. Although the Pearson correlation analysis result is reliable, it can have false positives due to the influence of several variables such as the magnitude of the slope at which the points are clustered and the heteroscedasticity [23], and thus results of RNA-seq analysis and qRT-PCR may be different. Therefore, verification of qRT-PCR of RNA-seq data is necessary.

As a result of co-expression network analysis, PPI network analysis, and functional annotation analysis, we found that genes and biological terms related to immune response, inflammatory responses, cytokine, and chemokine activity were enriched in both modules, and TNFα and NF-κB signaling-related genes were also found in both modules. Interestingly, in terms of inflammatory response, the large turquoise module was enriched with interferon signaling-related functions, and the small cyan module was enriched with the CXC chemokine in particular. Regarding cell proliferation, survival, and metabolism, the large turquoise module was associated with negative regulation of metabolic processes, and positive regulation of apoptotic processes such as TRAIL signaling and necroptosis. On the other hand, the small cyan module showed positive regulation of metabolic processes and more enriched MAPK signaling regulation including activated tak1 mediates p38 mapk activation compared to the large turquoise module.

As summarized above, TNFα-related genes (including also some TNF family members themselves) and NF-κB-related genes (including also NF-κB components) are present in both modules. As such, this is in line with the known roles of the TNFs and NF-κB as global regulators of inflammation, i.e. as regulators of multiple aspects of inflammation. However, the response of the TNFα- or NF-κB-related genes to JQ1 is different between the two modules, such that the TNFα- or NF-κB-related genes are downregulated along with the IFN-related genes in the large turquoise module but upregulated or unchanged along with the CXC chemokine genes in the small cyan module. Hence, different aspects of inflammation regulated by TNFs or NF-κB respond differently to JQ1, hence are under distinct epigenetic controls. Given that the TNFs and NF-κB are each related to more than one signaling pathway [24], it will be of interest to relate the large turquoise vs. small cyan modules to those specific pathways.

A complementary message emerges from the findings that we made with the more specialized inflammatory regulators, namely the IFNs and CXC chemokines. The direct biological effects of IFNs (mediated mainly by JAK/STAT signaling) are related especially, albeit not exclusively, to the modulation of immune system [25]; the direct biological effect of CXC chemokines is especially, albeit not exclusively, to promote neutrophil migration [26].

Here, we found that along with the TNFα/NF-κB genes of the large turquoise module, the IFN (mainly type I and II)-related genes (including IFNB1), which were limited to that same module, were also inhibited by JQ1. This agrees with the literature, since the direct inhibition of IFN response by BET inhibitors has been well documented [27]. Furthermore, we found that along with the TNFα/NF-κB-related genes of the small cyan module, the CXC chemokine genes (encoding CXCL1/2/3/5/8, all known to bind the chemokine receptor CXCR2), which were limited to that same module, were not inhibited by JQ1. We note in this respect that the GO term “activated tak1 mediated p38 mapk signaling activation” was also enriched in the small cyan module, in line with the known role of tak1/p38 signaling in CXC chemokine signaling [28]. Thus, not the type of cytokine per se (TNF vs. IFN vs. CXC chemokine) determines the outcome of JQ1 treatment, but the belonging to the large turquoise vs. small cyan module.

The selectivity of the gene response to I-BET has been reported to be related to the epigenetic status of the responding gene and the mechanism of BET recruitment. Nicodeme E et al. reported that some LPS-induced cytokines and chemokines, such as Tnf, Ccl2-5 and Cxcl1/2, have a highly selective effect on I-BET in bone marrow-derived macrophages. Further study on the mechanism of selectivity for BET inhibitors is needed, and considering the previous reports, selectivity for BET inhibitors may be related to the BET recruitment pathway or histone acetylation level for each gene, and the binding of the BET protein to the gene promoter or super-enhancer.

Of note, the DElncRNAs that we identified in the LPS-stimulated HMC3 cells have been observed in other inflammation-related contexts before. IRF1-AS1 (Lnc-SLC22A5-6) was found to act as a positive modulator of the IFN response in esophageal squamous cell carcinoma (ESCC), functioning as a tumor suppressor by regulating cell proliferation and apoptosis [29]. MIR155HG was increased in human M1 (inflammatory-type) macrophages that were derived from monocytes by treatment with LPS and IFN-gamma [30]. In human B cell lymphoma cells, LPS induced the nuclear translocation of an NF-κB p50/p65 heterodimer that could bind to the MIR155HG promoter, suggesting that MIR155HG was a direct NF-κB target gene [31]. MIR3142HG, the host gene for miR-3142 and miR-146a, was reported to regulate the IL-1β-induced inflammatory response in idiopathic pulmonary fibrosis (IPF) [32] and to be highly expressed in LPS-exposed human pulmonary microvascular endothelial cells (HPMECs) [33]. It is therefore of interest that our study showed that the LPS-induced increase of MIR3142HG was reduced by JQ1. However, the expression levels of MIR3142HG-correlated genes are controversial. In our study, JQ1 was not or only marginally effective on the MIR3142HG-correlated genes. Finally, LINC02605 (IL7-AS; Lnc-ZC2HC1A-1) was reported to regulate the immune response in a cell-type specific manner [34]. IL7-AS is a positive regulator of the IL1β-induced inflammatory response in human A549 epithelial cells, but a negative regulator in LPS-stimulated human THP-1 monocytes and mouse RAW 264.7 macrophages. Additionally, knockdown of IL7-AS increased the IL-6 release in IPF-derived fibroblasts, indicating that it is a negative regulator [32]. Our co-expression network analysis showed that while LINC02605 was induced by LPS, neither LINC02605 nor its correlated genes were significantly affected by JQ1. Interestingly, this was observed for all three DElncRNAs and their correlated genes in the small cyan module. These results are consistent with our previous transcriptome analyses of human and mouse microglial cells and mouse bone marrow-derived macrophages [8–10].

## Conclusion

Here, we present a comprehensive analysis of inflammation-related DElncRNA and DEmRNA expression profiles and functional networks in the human microglial cell line HMC3. We identified 5 DElncRNAs (including 4 novel ones in microglia) and 99 DEmRNAs. We constructed DElncRNA-DEmRNA co-expression networks, which fell into two separate modules, and investigated their functions and pathways, which – for both modules—turned out as largely known to be inflammation-related. We determined that although considered as an anti-inflammatory agent, the BET inhibitor JQ1 regulates the two modules differently, showing an almost uniform anti-inflammatory effect on one module, but little or even enhancing effect on the other. This interesting result will have to be complemented and validated with methods such as knockdown and overexpression; it is also of interest whether it can be extended to other models of neuroinflammation. Altogether, the RNA expression modules that we identified here provide a resource for further studies of human microglial neuroinflammation through both computational analysis and functional approaches.

## Materials and methods

### Identification of differentially expressed lncRNAs and mRNAs

For this study, we used the RNA-seq data of our previous paper (GSE155408). To identify DElncRNAs, a comprehensive reference list of known lncRNAs was included in the processing of the RNA-seq data [10, 35, 36]. Briefly, FASTQ data were quality controlled and trimmed with Trimmomatic (version 0.36) [37]. The FASTQ files were aligned using STAR (version 2.7.8) [38] alignment software with the GENCODE Homo sapiens reference sequence GRCh38 (Release 27). DElncRNAs and DEmRNAs were normalized to sequencing depth and RNA composition using the median method with default parameters of DESeq2 [39]. Differential expression analysis of lncRNA and mRNA was conducted using the DESeq2 R package. The DElncRNAs and DEmRNAs were selected with a cutoff of | log_2_ fold change (log_2_ FC) | ≥ 1.2, | log_2_ FC | ≤ -1.2, and adjusted *p*-value (*p*adj) ≤ 0.05 in LPS-treated HMC3 cells.

### Weighted gene co-expression network analysis (WGCNA)

First, the Pearson correlation coefficient (*r*) values were calculated to assess the similarity of the expression patterns of transcripts. Then, a scale-free network was obtained by weighting the correlation coefficient between transcripts with soft-thresholding power. A module is defined as a cluster of densely interconnected transcripts in terms of co-expression. We considered a |*r|*≥ 0.75 as a meaningful value. Cytoscape MCODE plug-in (Version 3.4.0, available online: http://www.cytoscape.org/) [40] was applied for visualization of the co-expression networks.

### Functional annotation and canonical pathway analysis

Database for Annotation, Visualization, and Integrated Discovery (DAVID, version 6.8) software (http://david.abcc.ncifcrf.gov/home.jsp) was used to analyze the biological functions in the datasets [41]. DAVID uses a modified Fisher's exact *p-*value to examine gene ontology (GO) enrichment. A false discovery rate (FDR) ≤ 0.05 was used as the criterion for GO term analysis. The KEGG Orthology Based Annotation System (KOBAS, version 3.0) software (http://kobas.cbi.pku.edu.cn/) [42] was used to analyze the enriched KEGG pathways [43] in the datasets. FDR ≤ 0.05 was used as the criterion for KEGG pathway enrichment analysis.

### Cell culture and treatment

HMC3 human microglial cells were purchased from the Korean Cell Line Bank (Seoul, Korea). The cells were cultured in minimum essential medium (MEM) supplemented with 10% fetal bovine serum (FBS), 100 IU/ml penicillin, and 10 μg/ml streptomycin and were maintained in a humidified incubator at 37 °C with 95% air/5% CO_2_. The cells were treated with 100 ng/ml LPS (Sigma-Aldrich, St. Louis, MO, USA) and/or 500 nM JQ1 for 4 h under standard culture conditions. The LPS and JQ1 were dissolved in dimethyl sulfoxide (DMSO; Sigma-Aldrich, St. Louis, MO, USA).

### Quantitative RT-PCR

Total RNA extractions and cDNA preparation were performed according to the manufacture’s instruction (Takara, Shiga, Japan). Quantitative Reverse Transcription PCR (qRT-PCR) was performed using an ABI 7500 real-time PCR system (Applied Biosystems Inc., Foster City, CA, USA). The critical threshold (△CT) value was normalized by the expression of an internal control, glyceraldehyde-3-phosphate dehydrogenase (GAPDH). Finally, the results were also analyzed using the comparative critical threshold (△△CT) method. The primers were designed using Primer Bank (http://pga.mgh.harvard.edu/primerbank/index.html) and are listed in Supplementary Table 4.

### Protein–protein interaction (PPI) network analysis

The Search Tool for the Retrieval of Interacting Genes (STRING, http://string.embl.de/) [44] was used to construct the PPI network for DEmRNAs (minimum required interaction score > 0.7). The interaction relationships of the proteins encoded by DEmRNAs were searched by STRING online software, and the combined score > 0.7 was used as the cut-off criterion. Cytoscape MCODE plug-in (Version 3.4.0, available online: http://www.cytoscape.org/) was applied for visualization of the protein–protein interaction. Additionally, the network analyzer was used to compute the basic properties of the PPI network, including average clustering co-efficient distribution, closeness centrality, average neighborhood connectivity, node degree distribution, shortest path length distribution, and topological coefficients.

### Statistical analysis

All data are expressed as the mean ± standard deviation of the mean (SD). The chi-square test was performed to confirm whether there was a statistically significant relationship between categorical variables by JQ1 treatment. A *p*-value or *p*adj ≤ 0.05 was considered significant. The statistical analyses were performed using IBM SPSS Statistics ver. 26.0 (IBM Corporation, Armonk, NY, USA). All qRT-PCR data were tested using one-way ANOVA followed by Tukey's honestly significant difference (HSD) post hoc test. Differences for which *p* ≤ 0.05 were considered significant.

## Supplementary Information

**Additional file 1: Supplementary Fig. 1.** Identification and functional annotation of DEmRNAs.

**Additional file 2: Supplementary Fig. 2.** qRT-PCR validation of the DEmRNAs.

**Additional file 3: Supplementary Table 1.** All significantly up- and downregulated DElncRNAs and DEmRNAs.

**Additional file 4: Supplementary Table 2.** Pearson correlation coefficient (r) values of DElncRNA-DEmRNA pairs.

**Additional file 5: Supplementary Table 3.** Results of the chi-square test for each module.

**Additional file 6: Supplementary Table 4.** List of primers used for qRT-PCR.

## Data Availability

The RNA-seq data used and analyzed during the current study are included in this published article and its supplementary files. The open RNA-seq data (Accession number: GSE155408) is available in the NCBI database (https://www.ncbi.nlm.nih.gov/geo/query/acc.cgi?acc=GSE155408).

## Abbreviations

BET: Bromodomain and extra-terminal
BP: Biological process
BRD: Bromodomain-containing protein
CC: Cellular component
DAVID: Database for annotation, visualization and integrated discovery
DEmRNA: Differentially expressed mRNA
DElncRNA: Differentially expressed lncRNA
FDR: False discovery rate
GO: Gene ontology
KEGG: Kyoto encyclopedia of genes and genomes
LncRNA: Long non-coding RNA
Log_2_ FC: Log_2_ fold change
LPS: Lipopolysaccharide
MF: Molecular function
PPI: Protein–protein interaction
qRT-PCR: Quantitative reverse transcription-polymerase chain reaction
RNA-seq: RNA sequencing
STRING: Search tool for the retrieval of interacting genes
WGCNA: Weighted gene co-expression network analysis
