# Supplementary material for: Analysis of differentially expressed long non-coding RNAs in LPS-induced human HMC3 microglial cells
Source: BMC Genomics. 2022 Dec 27;23:853. doi: 10.1186/s12864-022-09083-6 (PMC9795738; doi:10.1186/s12864-022-09083-6)
Supplement: Supplementary file 1 — Additional file 1: Supplementary Fig. 1. Identification and functional annotation of DEmRNAs. [file 12864_2022_9083_MOESM1_ESM.docx]

**
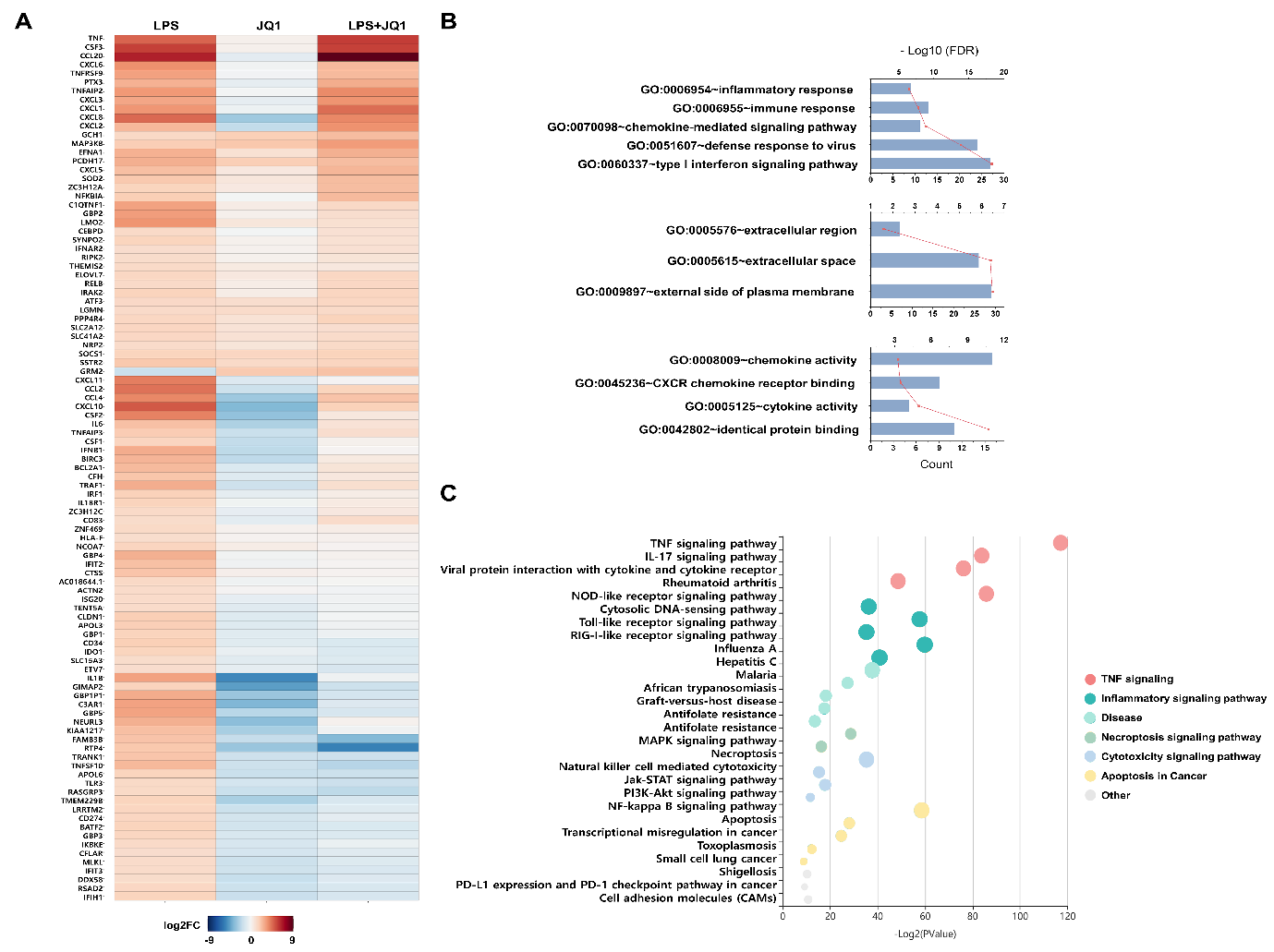
**

**Supplementary Fig. 1. Identification and functional annotation of DEmRNAs.**

(A) Heat map showing the expression changes of the 5 LPS-induced mRNAs that qualified as DEmRNAs by the criteria of this study (see Materials and methods), and the heat maps for the same DElncRNAs upon treatment with JQ1 or LPS + JQ1. The color scale represents the log_2_FC values. Shown are the GO term and KEGG pathway enrichment analyses of the total DEmRNAs. (B) In the GO term analyses, the top 5 GO terms are displayed in BP (upper panel), CC (middle panel), and MF (bottom panel). The blue column is the count value indicating the number of genes enriched in the GO term, and the red line is the -log_10_ (FDR) value. (C) In the KEGG pathway enrichment analyses, each row represents an enriched function, and the size of the bubble represents the *p* value (KOBAS, http://kobas.cbi.pku.edu.cn). The KOBAS algorithm divides the clusters according to the values computed for the enriched pathway, and the color of each bubble represents a different cluster.
