# Supplementary material for: Analysis of differentially expressed long non-coding RNAs in LPS-induced human HMC3 microglial cells
Source: BMC Genomics. 2022 Dec 27;23:853. doi: 10.1186/s12864-022-09083-6 (PMC9795738; doi:10.1186/s12864-022-09083-6)
Supplement: Supplementary file 2 — Additional file 2: Supplementary Fig. 2. qRT-PCR validation of the DEmRNAs. [file 12864_2022_9083_MOESM2_ESM.docx]

**
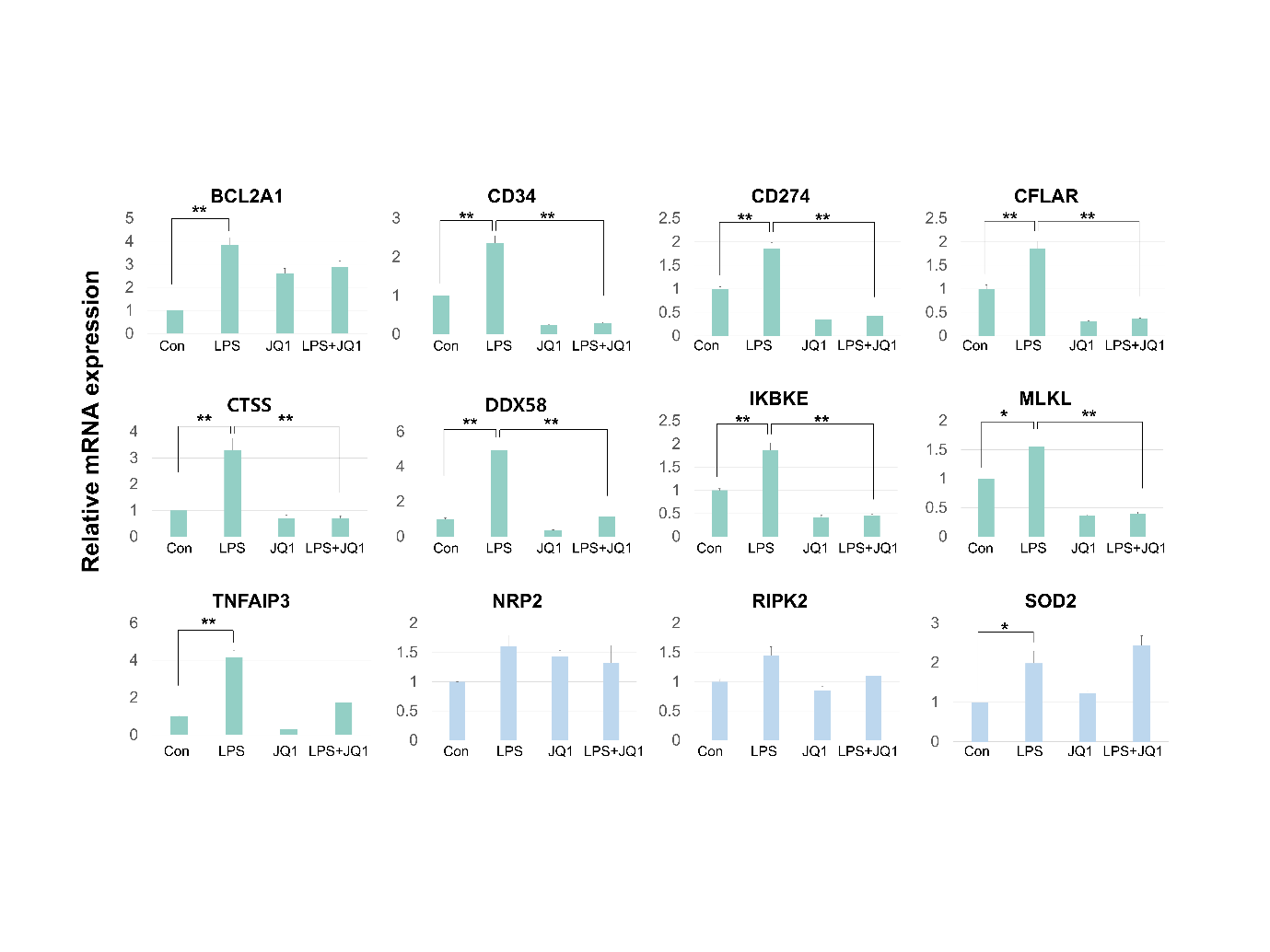
**

Con

LPS+JQ1

JQ1

LPS


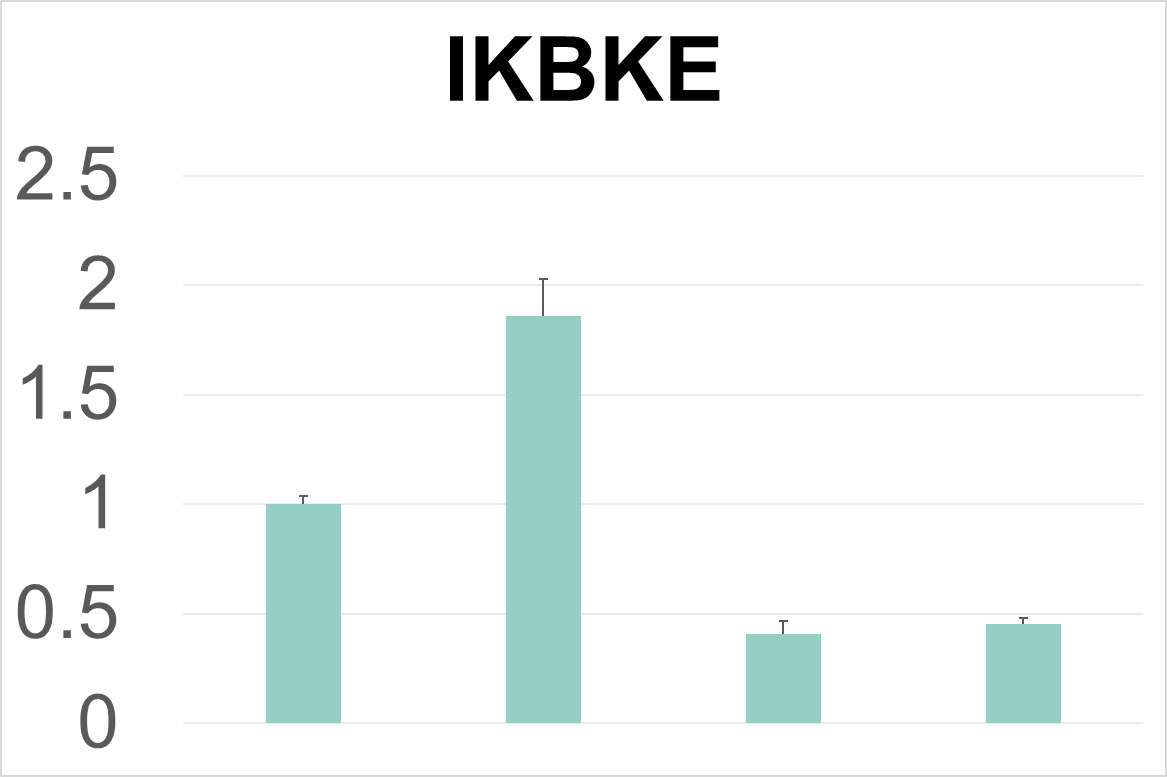


******

******


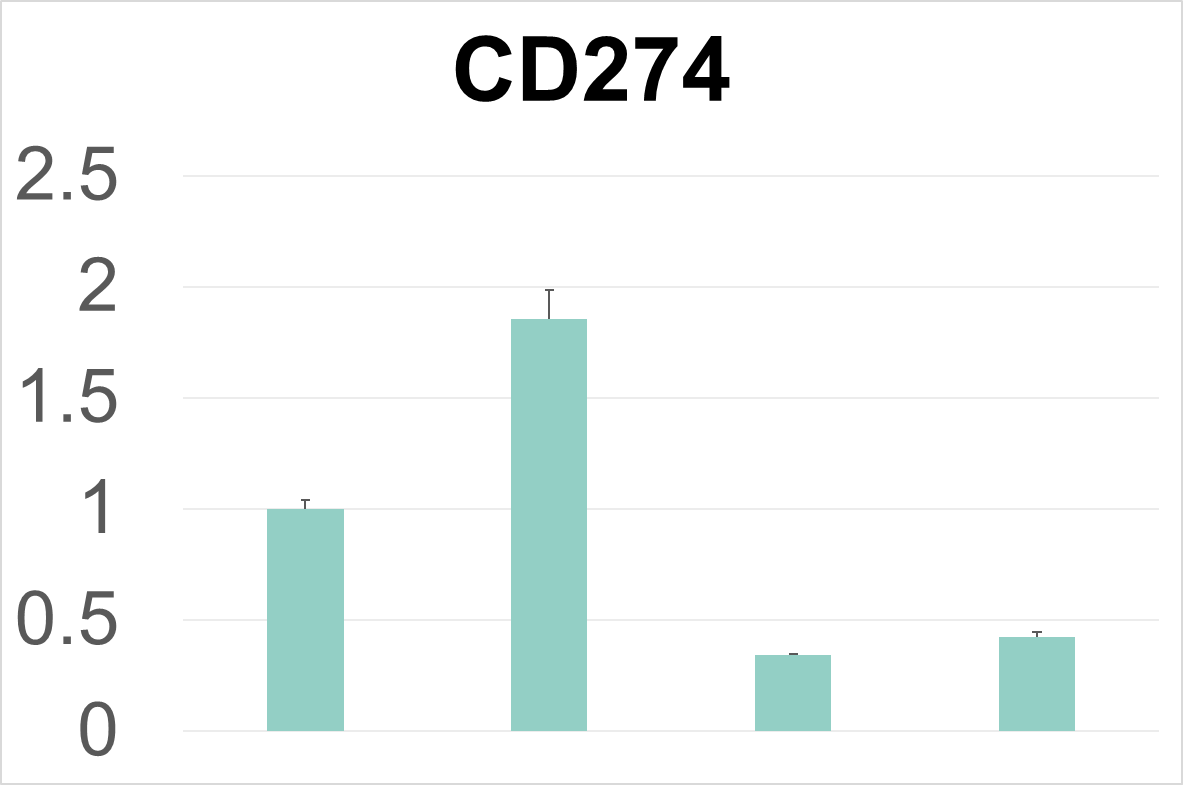


Con

LPS+JQ1

JQ1

LPS

******

******

Con

LPS+JQ1

JQ1

LPS


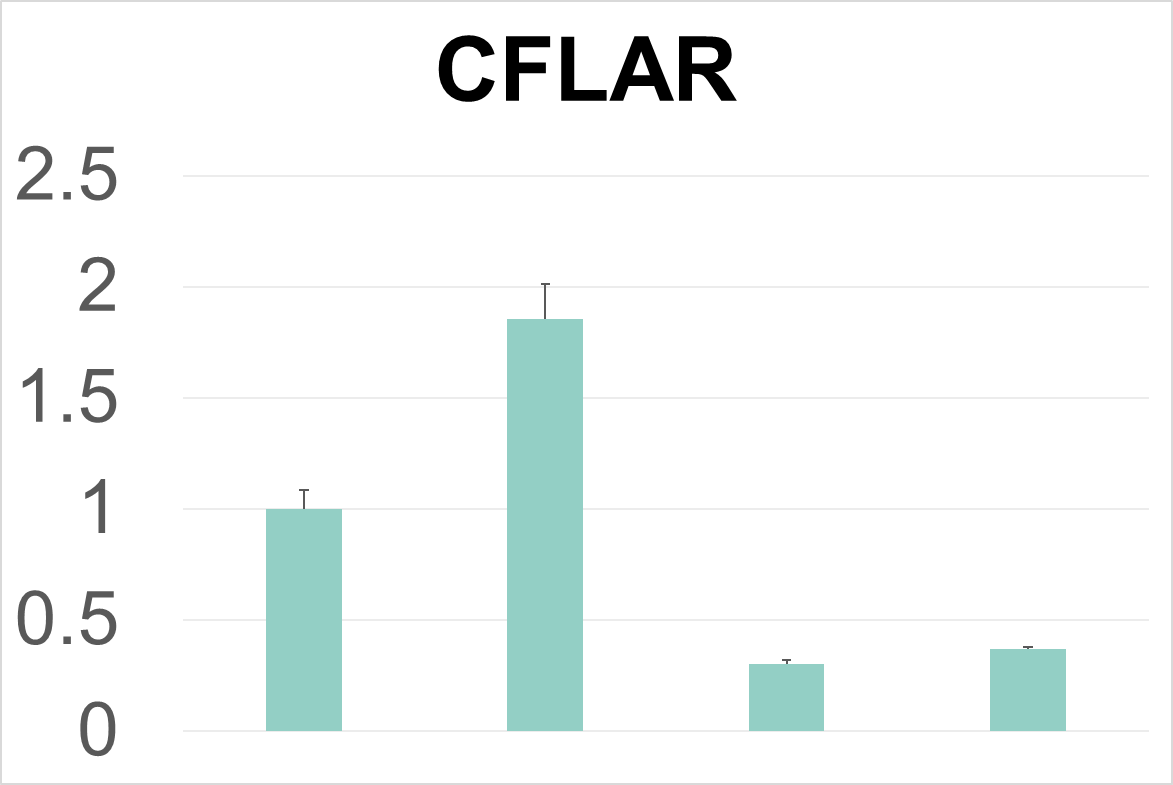


******

******

Con

LPS+JQ1

JQ1

LPS


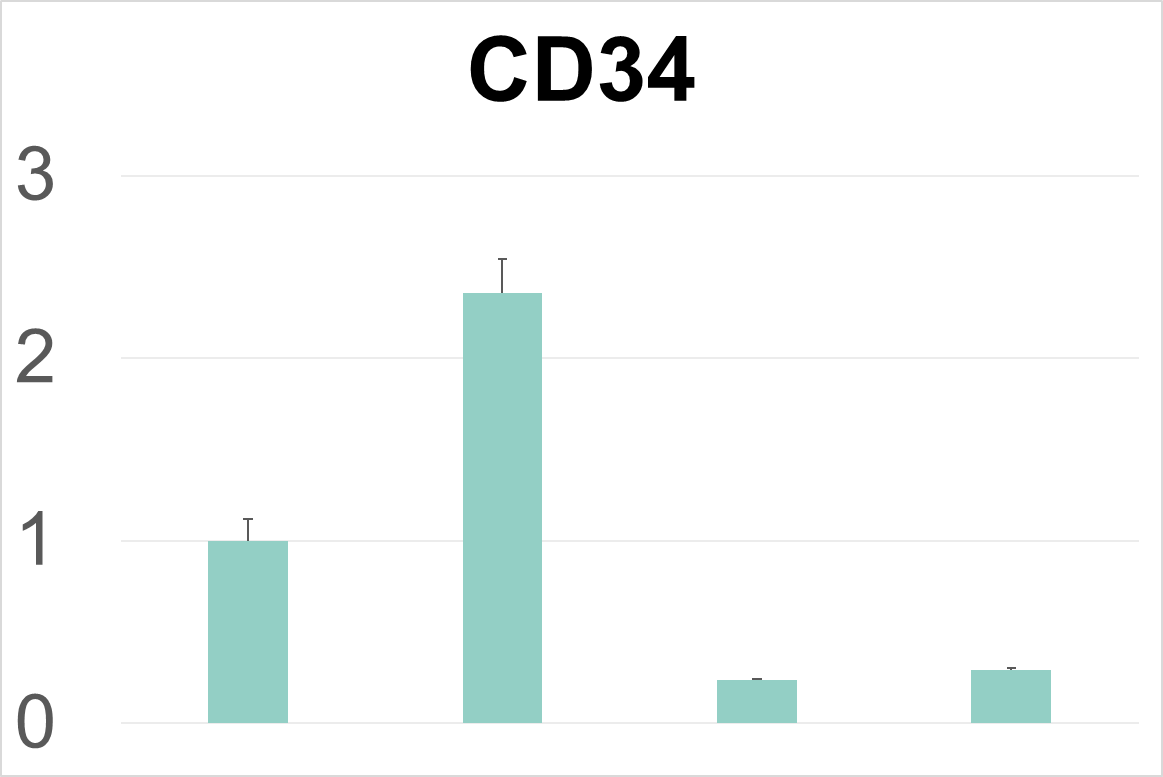


******

******

Con

LPS+JQ1

JQ1

LPS


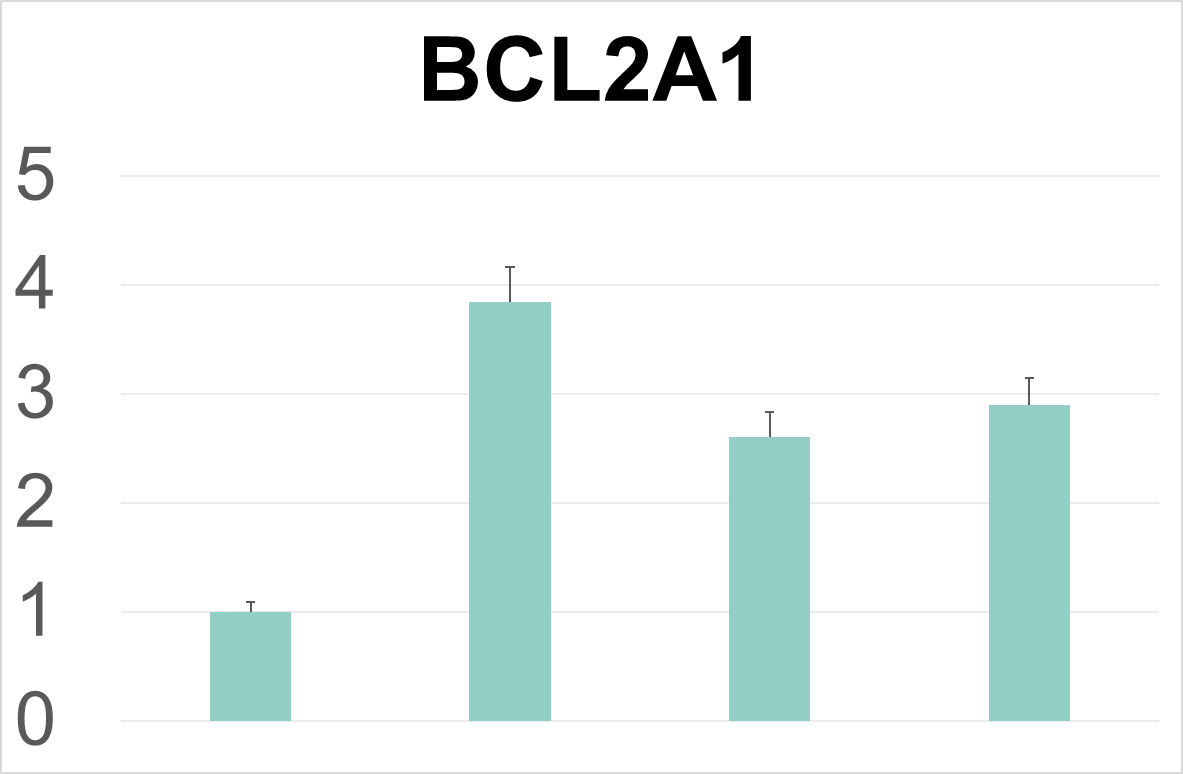


******


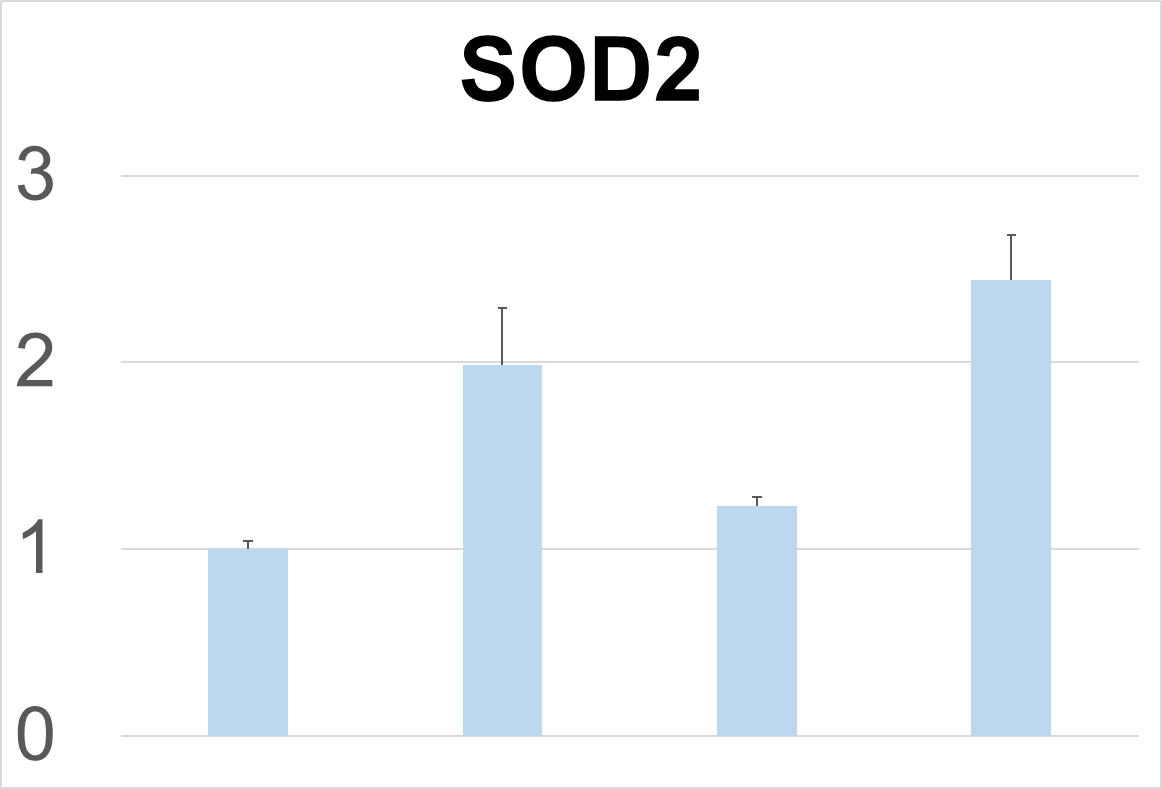


Con

LPS+JQ1

JQ1

LPS

*****

Con

LPS+JQ1

JQ1

LPS


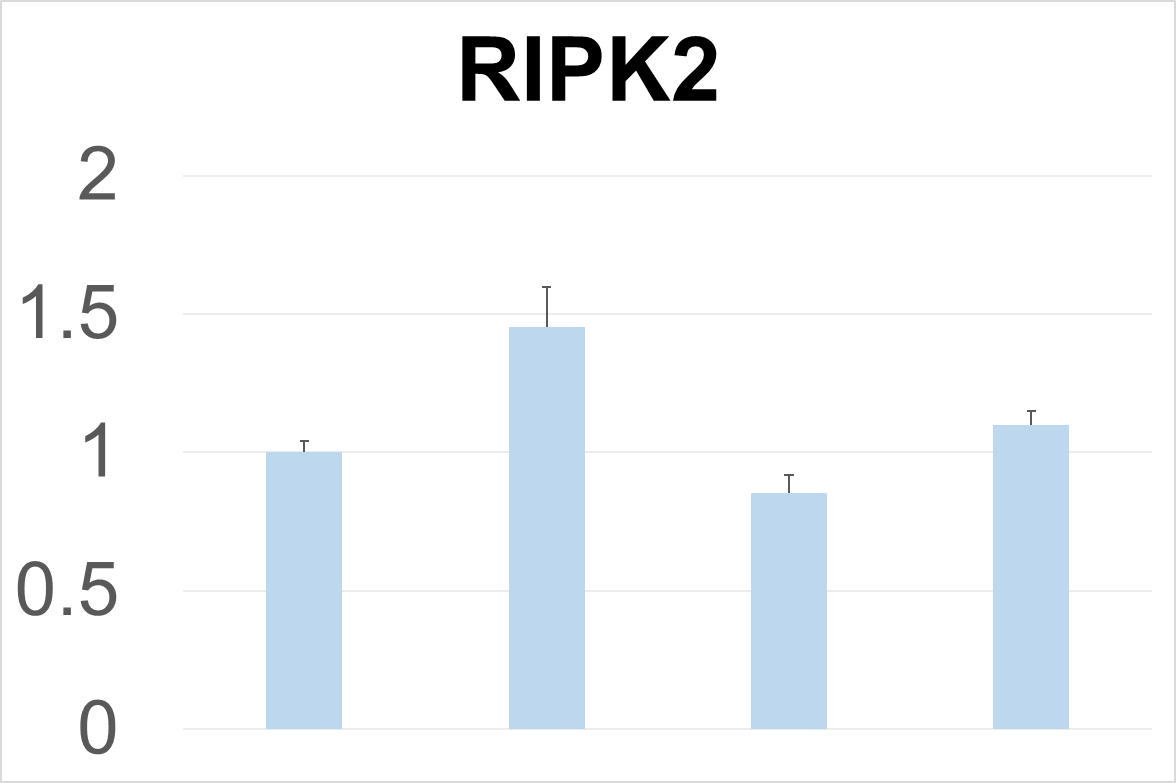

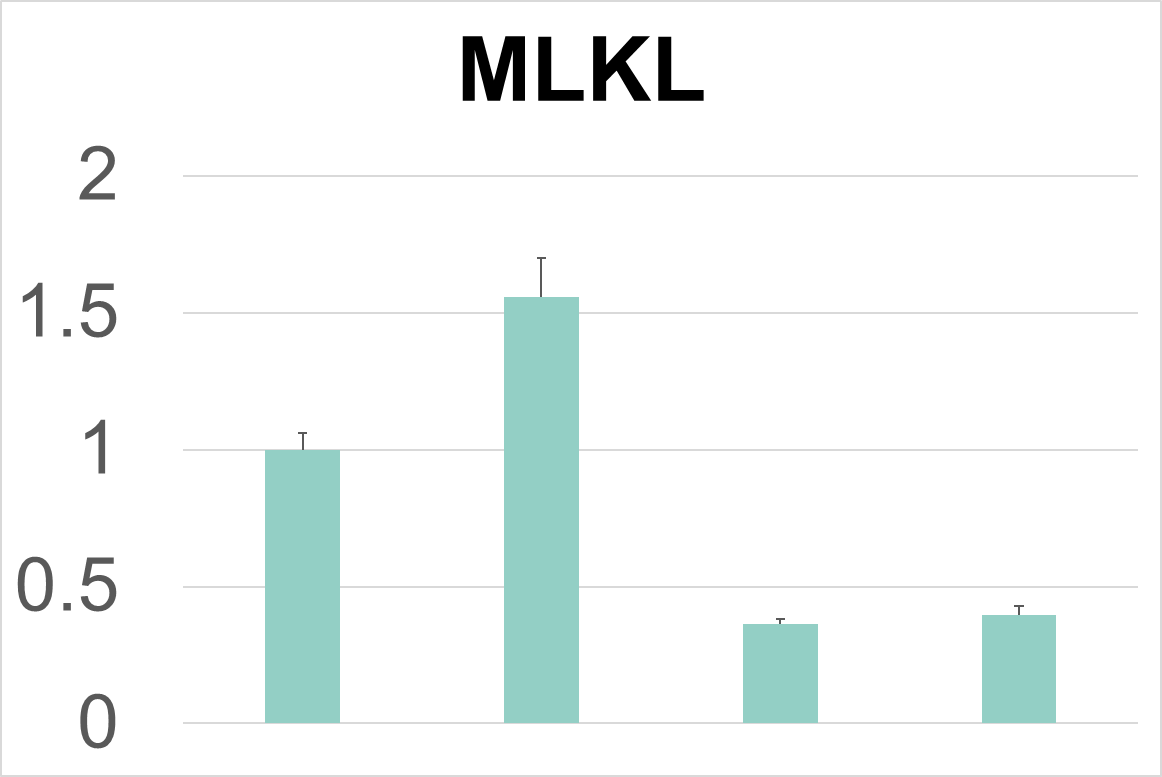


Con

LPS+JQ1

JQ1

LPS

*****

******


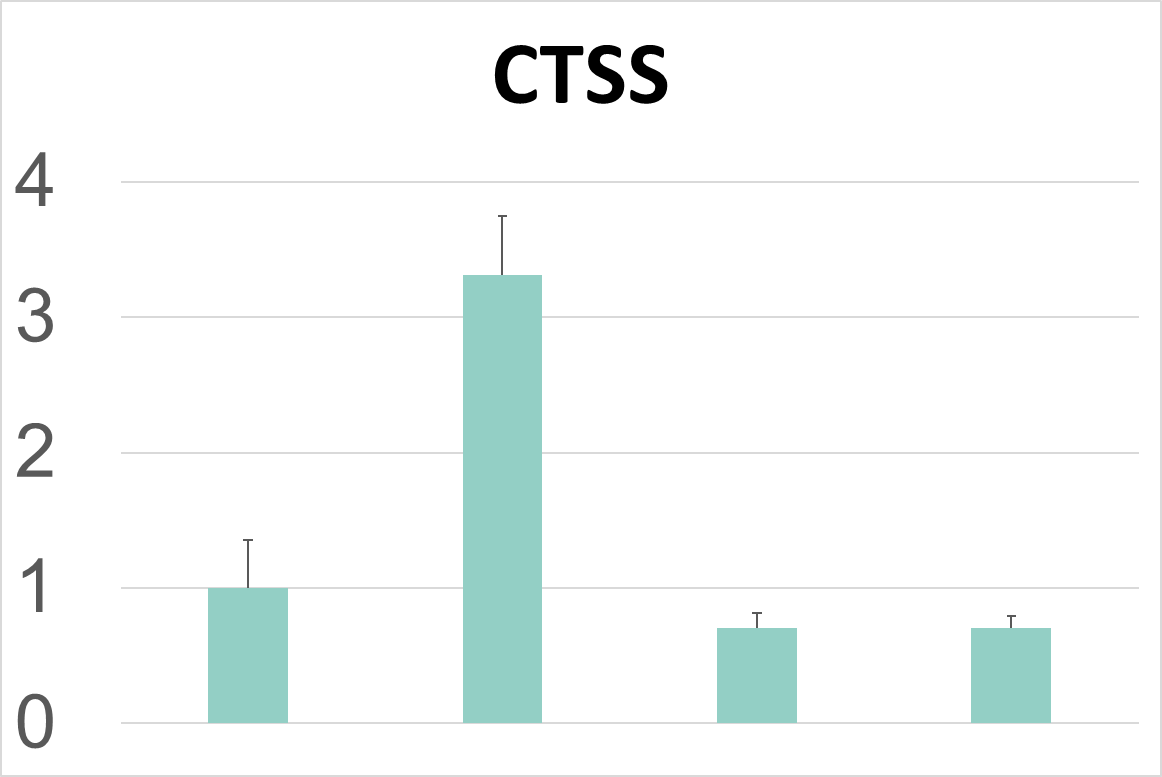


Con

LPS+JQ1

JQ1

LPS

******

******


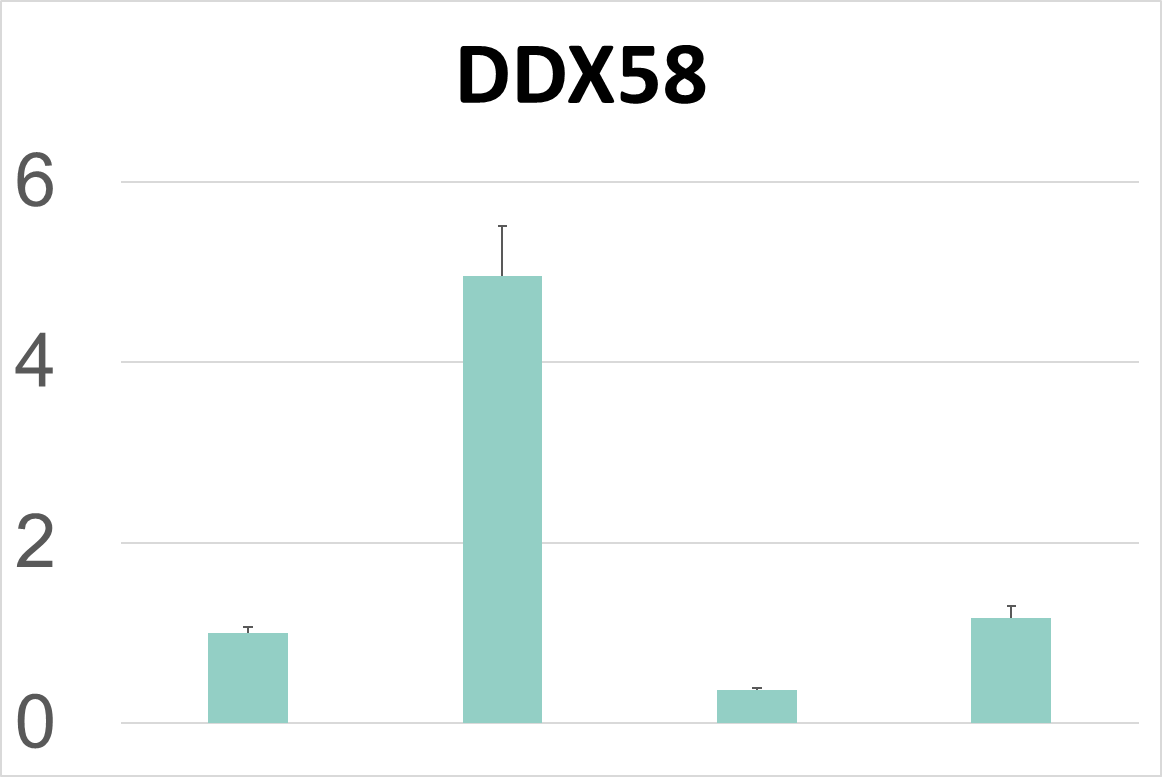


Con

LPS+JQ1

JQ1

LPS

******

******

**Relative mRNA expression**

**Supplementary Fig. 2. qRT-PCR validation of the DEmRNAs.**

Additional mRNAs were verified in HMC3 cells. Green indicates mRNAs of the large turquoise module, and blue indicates mRNAs of the small cyan module. The RNA levels are normalized to GAPDH transcript levels. The data represent three independent experiments. The values are the mean ± SEM of triplicate experiments (**p* < 0.05 and ***p* < 0.001).
